# Primary ectocervical epithelial cells display lower permissivity to *Chlamydia trachomatis* than HeLa cells and a globally higher pro-inflammatory profile

**DOI:** 10.1038/s41598-021-85123-7

**Published:** 2021-03-12

**Authors:** Chongfa Tang, Chang Liu, Benoit Maffei, Béatrice Niragire, Henri Cohen, Aminata Kane, Anne-Claire Donnadieu, Yael Levy-Zauberman, Thomas Vernay, Juliette Hugueny, Etienne Vincens, Christine Louis-Sylvestre, Agathe Subtil, Yongzheng Wu

**Affiliations:** 1 Unité de Biologie cellulaire de l’infection microbienne, Institut Pasteur, UMR3691 CNRS, 75015 Paris, France; 2 National Vaccine and Serum Institute, Beijing, China; 3 Sorbonne Université, Collège doctoral, 75005 Paris, France; 4 Service de Chirurgie gynécologique, Institut Mutualiste Montsouris, 75014 Paris, France; 5 Service de Gynécologie, Clinique Saint Jean de Dieu, 75007 Paris, France

**Keywords:** Cytokines, Infection, Infectious diseases, Inflammation, Innate immune cells, Innate immunity, Mucosal immunology, Bacteria, Pathogens, Microbiology, Diseases, Infectious diseases, Urogenital diseases

## Abstract

The tumoral origin and extensive passaging of HeLa cells, a most commonly used cervical epithelial cell line, raise concerns on their suitability to study the cell responses to infection. The present study was designed to isolate primary epithelial cells from human ectocervix explants and characterize their susceptibility to *C. trachomatis* infection. We achieved a high purity of isolation, assessed by the expression of E-cadherin and cytokeratin 14. The infectious progeny in these primary epithelial cells was lower than in HeLa cells. We showed that the difference in culture medium, and the addition of serum in HeLa cultures, accounted for a large part of these differences. However, all things considered the primary ectocervical epithelial cells remained less permissive than HeLa cells to *C. trachomatis* serovar L2 or D development. Finally, the basal level of transcription of genes coding for pro-inflammatory cytokines was globally higher in primary epithelial cells than in HeLa cells. Transcription of several pro-inflammatory genes was further induced by infection with *C. trachomatis* serovar L2 or serovar D. In conclusion, primary epithelial cells have a strong capacity to mount an inflammatory response to *Chlamydia* infection. Our simplified purification protocol from human explants should facilitate future studies to understand the contribution of this response to limiting the spread of the pathogen to the upper female genital tract.

## Introduction

Infections of the reproductive system are a major global health concern and have become more critical recently due to the increase in incidence of sexually transmitted infections (STIs) with more than 1 million new cases per day worldwide, among which *Chlamydia trachomatis* infection is predominant^1^. Due to its anatomy and hormonal regimen, the female genital tract (FGT) is two to three times more likely than the male’s to be infected by certain microorganisms such as *Neisseria gonorrhea* and *Chlamydia trachomatis*^2,3^. Infections by the latter are asymptomatic in most cases. However, *C. trachomatis* can ascend from the lower to the upper genital tract of females, leading to pelvic inflammatory diseases, tubal infertility, ectopic pregnancy and even cancer^4^.

The surface of the FGT is covered with tightly packed epithelial cells that, together with the mucus that covers the outer surface, constitute a physical barrier between the external environment and the internal tissues. The lower FGT, i.e., the vagina and the ectocervix, is made of multiple layers of stratified epithelia, while a single layer of columnar epithelia covers the different parts of the upper FGT, i.e., the endocervix, uterus, fallopian tubes and ovaries^5^. The epithelial cells in the FGT are exposed to a variety of “foreign” cells: microorganisms of the vaginal flora, sexually transmitted pathogens, sperm, and during pregnancies, they have to tolerate the presence of semi-allergic fetus^6^. Together with stromal cells and other antigen presenting cells, epithelial cells present antigens to activate adaptive immunity of the host^7^.

*Chlamydia trachomatis*, an obligate intracellular bacterium, resides and develops mainly within epithelial cells of the FGT. Thus, epithelial cells, especially in the lower FGT, are at the front line of host defense against *C. trachomatis* infection. A better understanding of their intrinsic ability to limit *C. trachomatis* infection is required, and would be complementary to the data obtained from animal models. For this purpose, HeLa cells, an epithelial cell line derived from a cervical cancer, have been widely used. However, HeLa cells are tumoral cells with many chromosomal aberrations that make them very different from normal cells^8,9^. Having been passaged for several dozens of generations they are even very different from the originally isolated cells. Therefore, HeLa cells are not an ideal cell model to study the properties of epithelial cells of the FGT, and normal somatic epithelial cells should be used instead. Primary epithelial cells isolated from the human cervix have been used by different groups^10–12^. They demonstrated, for instance, the expression of Toll-like receptors by these cells^13^ and their capacity for antigen-presentation^14^. However, studies on their response to *Chlamydia* infection remain scarce^10,15–19^.

In the present study we isolated and expanded primary epithelial cells from cervical explants of donors undergoing hysterectomies, and compared the host inflammatory response to *C. trachomatis* infection of these primary epithelial cells to that of HeLa cells.

## Results

### Isolation and characterization of primary epithelial cells

Epithelial cells were isolated from ectocervical explants excised after surgery on women undergoing hysterectomy as described in the methodology section and illustrated in Fig. S1. The stromal tissue was removed as much as possible from the explant, and the remaining tissue containing the mucosal layer was cut into small pieces (Fig. S1a), which improved the efficacy of subsequent enzymatic digestion by Dispase II. To limit contamination with fibroblasts, we introduced after digestion a mechanical separation of the mucosal layer, that is made of epithelial cells and mucus, from the stromal tissue (Fig. S1b). Dispase II digestion for either 3–4 h at 37 °C or overnight at 4 °C gave similar results and allowed a good separation of the mucosal layer in > 90% of the cases. The mucosal layers were then treated with trypsin and EDTA to dissociate the epithelial cells (Fig. S1c,d). We observed that a vigorous agitation after the trypsin/EDTA treatment improved cell dissociation. Non-dissociated tissue and debris were removed by filtration (Fig. S1e), and the isolated cells (Fig. S1f) were transferred into K-SFM.

**Figure 1:**
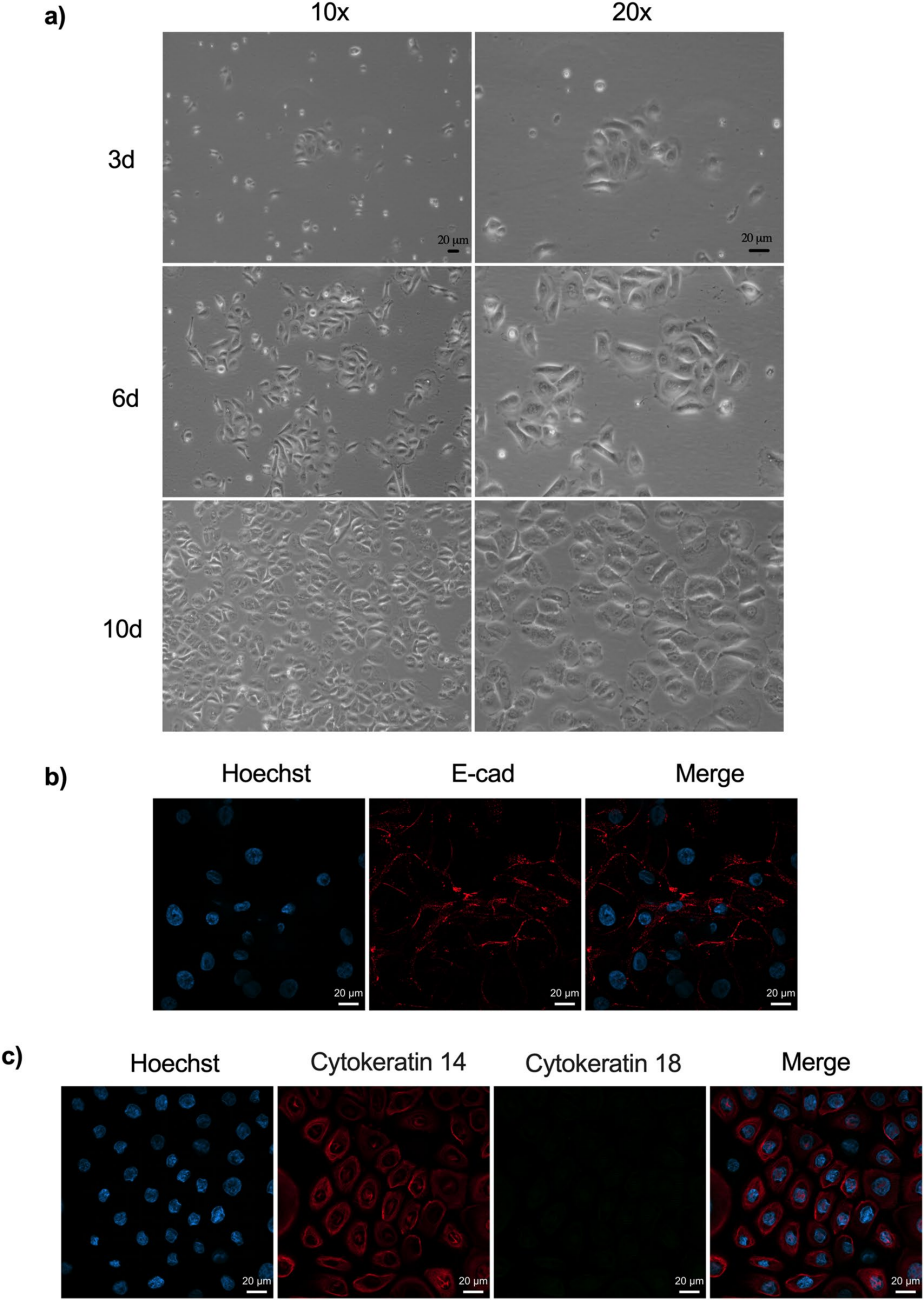

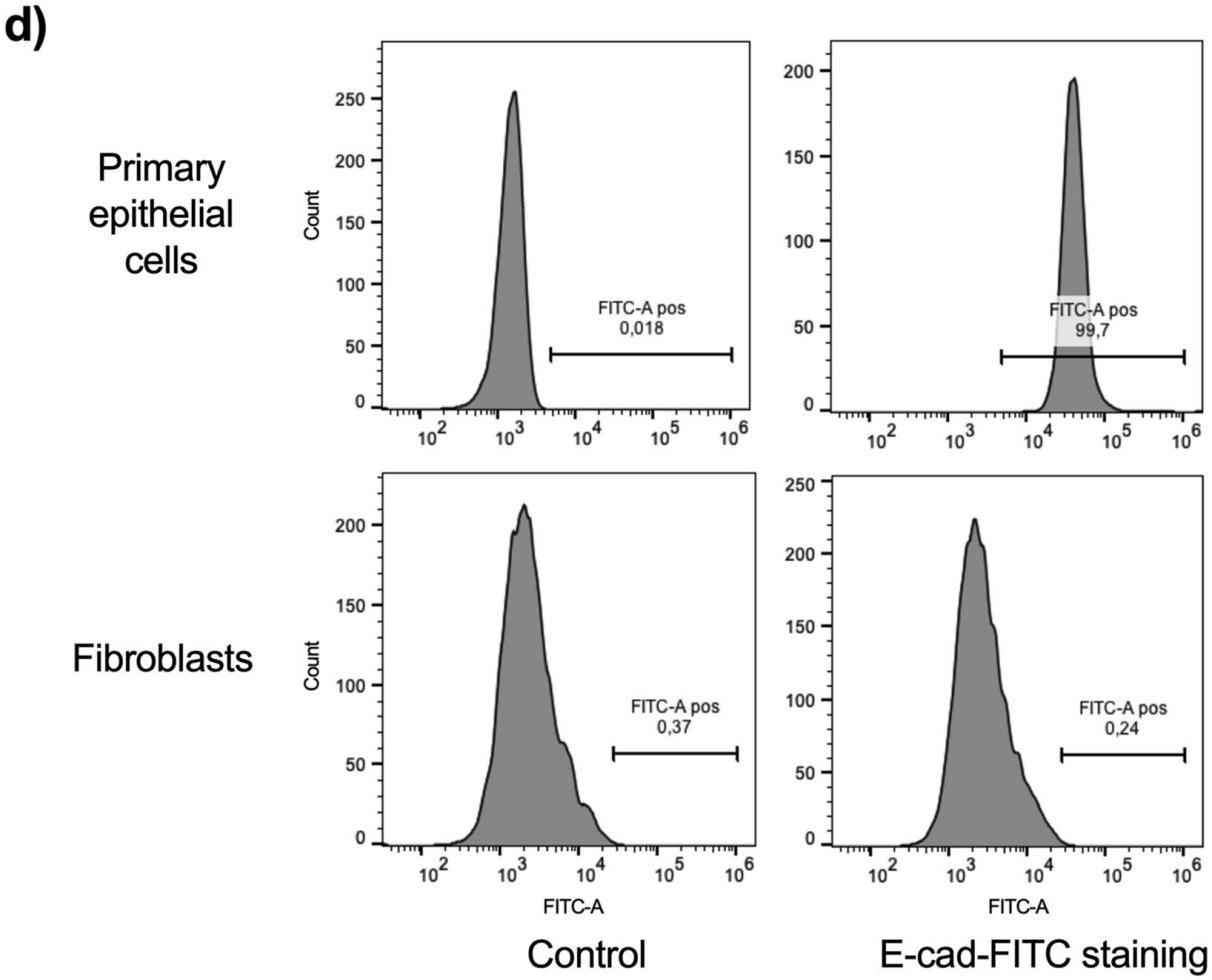
Growth and characterization of primary epithelial cells. (**a**) Contrast phase images were taken 3, 6 and 10 days after seeding the cells. (**b**,**c**) Cells seeded were fixed and stained with anti-human E-cadherin (**b**) or with anti-human cytokeratin 14 or 18 (**c**) antibodies and fluorochrome-conjugated secondary antibodies. DNA was labeled with Hoechst. (**d**) Epithelial cells and fibroblasts were isolated from one explant and stained (right) or not (left) with FITC-conjugated anti-human E-cadherin antibody, and analyzed by flow cytometry. All experiments were performed using primary epithelial cells isolated from at least 3 donors.

Isolated cells were seeded directly in 75 cm^2^ culture flask. Signs of cell divisions were apparent 3 days after seeding, with regular occurrence of small colonies (Fig. 1a). Around 10 days after seeding, the cells (passage 1) showed homogenous morphology, with no detectable contamination by other cell types such as fibroblasts and stromal cells, based on morphological features. We stained the cells with an antibody against E-cadherin, a specific marker of epithelial cells. Observation under a fluorescence microscope showed a plasma membrane localization of E-cadherin on the large majority of the cells, as expected (Fig. 1b). This was confirmed by quantification by flow cytometry, which detected positive E-cadherin signal in > 95% of the cells (Fig. 1d). Primary fibroblasts isolated from the same donor’s tissue served as negative control in that experiment (Fig. 1d). Consistent with the observations that ectocervical epithelial cells express cytokeratin 14 but not 18^20,21^, we observed only cytokeratin 14 in the isolated primary cells (Fig. 1c). These results, together with our morphological observations, showed that the large majority of the isolated cells were epithelial cells.

### Culture in ‘defined K-SFM’ reduced contamination by fibroblast

Although most passage 1 cultures consisted almost exclusively of epithelial cells, we often observed the emergence of elongated cells, i.e. the typical morphology of fibroblastic cells, upon later passages, or upon thawing cells that had not shown signs of fibroblast contamination at the time of freezing. To solve this problem, we tested a newly commercialized medium designed for the culture of primary epithelial cells, designated here as ‘defined K-SFM’. One vial of frozen primary epithelial cells (passage 1 or 2) conserved in liquid nitrogen was thawed and split into two cultures, one in K-SFM containing EGF and BPE (named ‘K-SFM’), one in defined K-SFM. Four days after culture, individual fibroblasts appeared in the K-SFM dish, and their relative proportion in the culture increased with time (Fig. S2a, upper panel). In contrast, no elongated cell was observed in the defined K-SFM dish, even 11 days after seeding (Fig. S2a, lower panel), suggesting that this medium inhibited fibroblast growth. To further verify this inhibitory effect on fibroblast proliferation, the sub-confluent cells from day 11 were collected and seeded in duplicate in new wells. Each duplicate well was incubated with either K-SFM or defined K-SFM (Fig. S2b). The two cultures in K-SFM medium showed gradual increase of fibroblasts contamination (Fig. S2c, 1st and 3rd rows). In contrast, the abundance of fibroblasts decreased significantly when K-SFM had been replaced by defined K-SFM (Fig. S2c, 2nd row) and cells continuously cultured in defined K-SFM showed almost no sign of fibroblast contamination (Fig. S2c, 4th row). This experiment showed that defined K-SFM markedly inhibited the growth of fibroblasts in the primary epithelial cell cultures.

### Primary epithelial cells isolated from human cervix were less permissive to *C. trachomatis* development than HeLa cells

**Figure 2:**
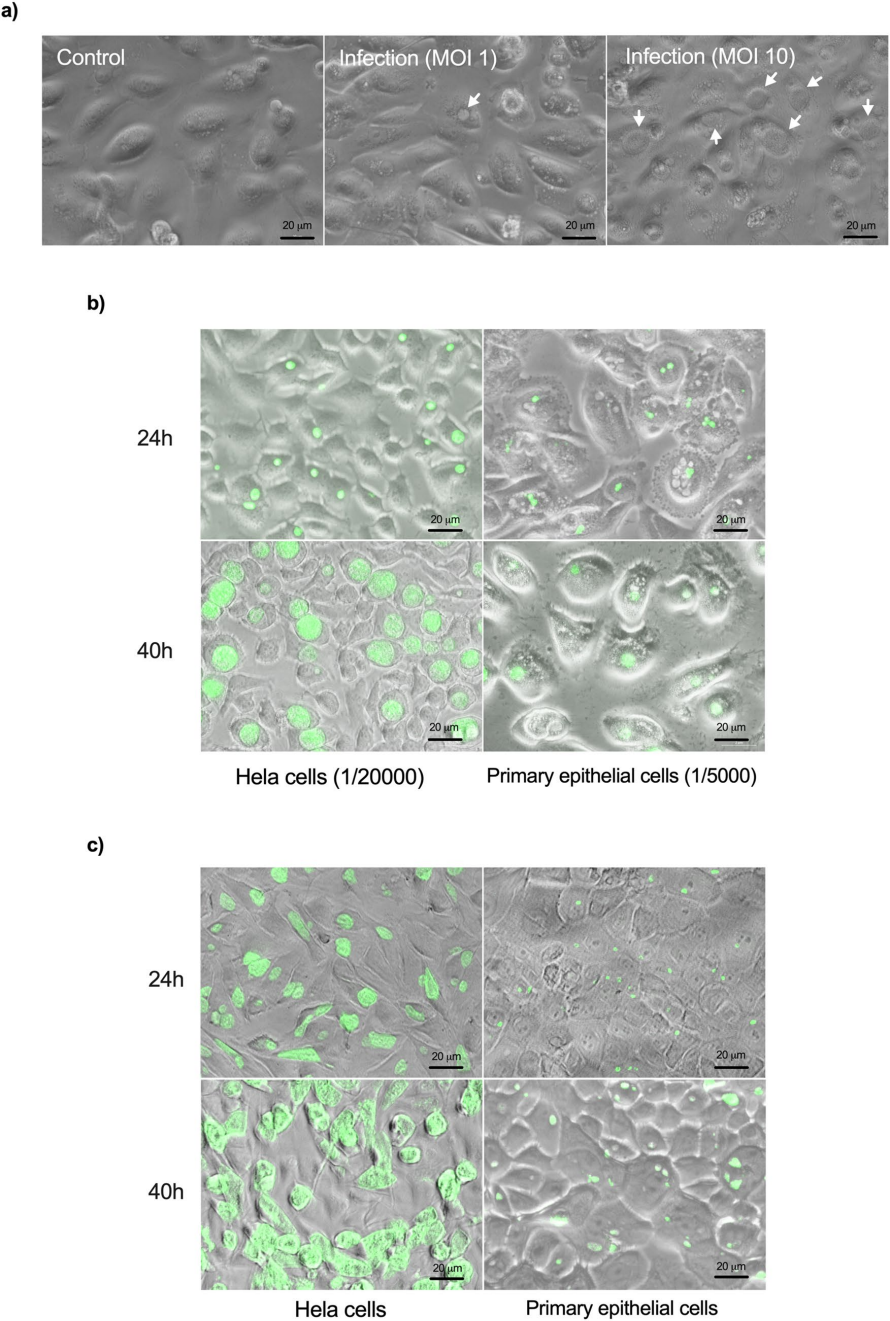

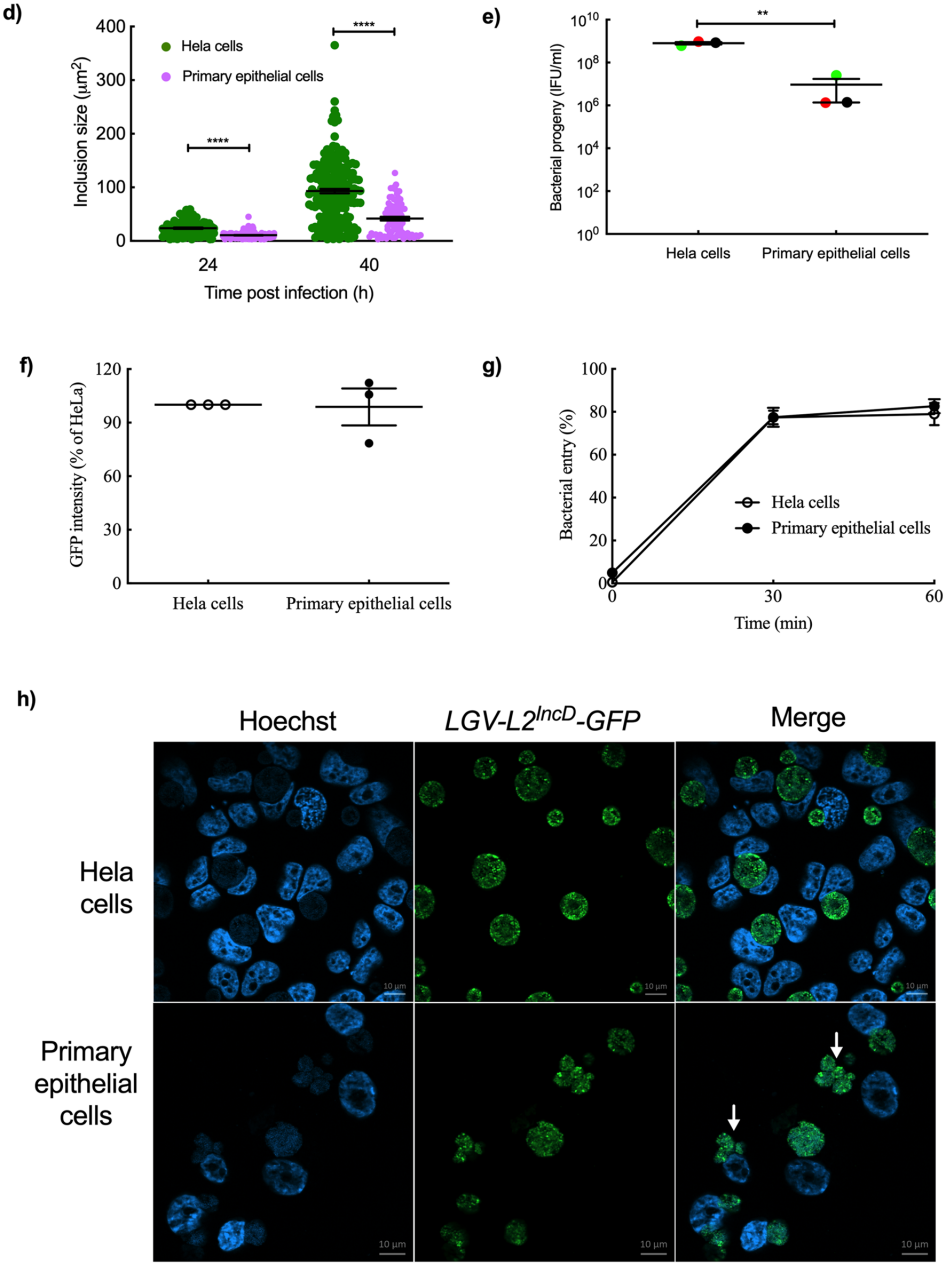
Primary epithelial cells are less permissive than HeLa cells to *C. trachomatis* development. (**a**) Primary epithelial cells were incubated with increasing quantities of bacteria and were observed 48 hpi. (**b**) Cells were infected with L2^incD^GFP (bacterial stock diluted 1:20,000 for HeLa cells and 1:5000 for primary cells) and observed 24 hpi (top) or 40 hpi (bottom). The picture displays merged images taken with phase contrast and in the green channel. (**c**) Cells were infected with *Chlamydia* serovar D (same quantity of bacteria used for both cell types) and observed 24 hpi (top) or 40 hpi (bottom). The cells were then fixed and stained with antibody against *Chlamydia* Cap1 and A488-conjugated secondary antibody. The picture displays merged images taken with phase contrast and in the green channel. (**d**) Quantification of inclusion sizes, each individual point represented one inclusion (n > 5 fields per experiment). (**e**) Titration of the progeny collected 48 hpi on HeLa cells and primary epithelial cells. (**f**) Cells were incubated for 4 h at 4 °C with L2^incD^GFP bacteria before measurement of attached bacteria by flow cytometry. (**g**) Cells were incubated with L2^incD^GFP bacteria for 30 min at 4 °C, before transfer to 37 °C. At the indicated times the cells were fixed and external bacteria were labeled with a red fluorochrome. The graph represented the number of internalized bacteria relative to total cell-associated bacteria. (**h**) Cells were incubated for 40 h with L2^incD^GFP bacteria. Arrows point to multiple inclusions, only observed in primary epithelial cells. The data were presented as mean ± SEM and the non-paired student’s t-test was used for two group comparisons. At least 2 independent were performed in (**a**–**c**) and (**h**), and primary cells used in each experiment were from more than one donor. (**e**–**g**) were from 3 independent experiments with primary epithelial cells from different donnors each time. **p < 0.01 and ****p < 0.0001.

We first checked whether isolated primary epithelial cells were permissive for *C. trachomatis* growth. Bacteria loaded vacuoles, called inclusions, were observed in infected cells and their numbers increased with the infectious dose, with > 90% infected cells at high dose (Fig. 2a). Given that the HeLa cell line is predominantly used by the *Chlamydia* community, we then compared the susceptibility to *C. trachomatis* infection in HeLa and primary epithelial cells. Same number of HeLa cells and primary epithelial cells were infected with serial dilutions of GFP-expressing bacteria (L2^incD^GFP). We observed that 4 to 10 times (depending on the donor sample) more bacteria were needed to reach an equivalent percentage of inclusion-bearing cells 24 h post infection (Fig. 2b). We also observed that the inclusions were smaller in primary epithelial cells compared to HeLa cells, both at 24 and 40 hpi (Fig. 2b,d). Consistent with these observations, a close to two-log difference in the infectious progeny collected 48 hpi (a duration corresponding on average to the completion of one cycle) was observed between the two cellular backgrounds (Fig. 2e). To test whether the binding efficiency of bacteria accounted for the decreased efficiency at establishing an infection observed in Fig. 2b, we measured the binding of L2^incD^GFP to HeLa and primary epithelial cells at 4 °C. No difference was observed (Fig. 2f), indicating that the low rate of infection of the primary epithelial cells was not due to defective binding of the bacteria. We next measured the efficacy of bacterial internalization, and again observed no difference between primary epithelial cells and HeLa cells (Fig. 2g, S3). These results indicate that the decrease in *C. trachomatis* infectivity in primary cells compared to HeLa cells were not due to defects in the attachment nor entry steps, but in failure, for about 75% to 90% of the bacteria, to initiate the development of a viable inclusion. Furthermore, the successful inclusions grew much slower, meaning that the bacteria divided less rapidly. All these observations were achived using a laboratory standard strain of *C. trachomatis* serovar L2 originally isolated from a man with proctitis. We then tested the susceptibility of primary epithelial cells to a *C. trachomatis* serovar D strain, one of the serovars commonly infecting the FGT. The serovar D strain also showed delayed development in primary epithelial cells compared to Hela cells (Fig. 2c, S4). Altogether, we concluded that primary epithelial cells were less permissive to *C. trachomatis* development than HeLa cells.

**Figure 3:**
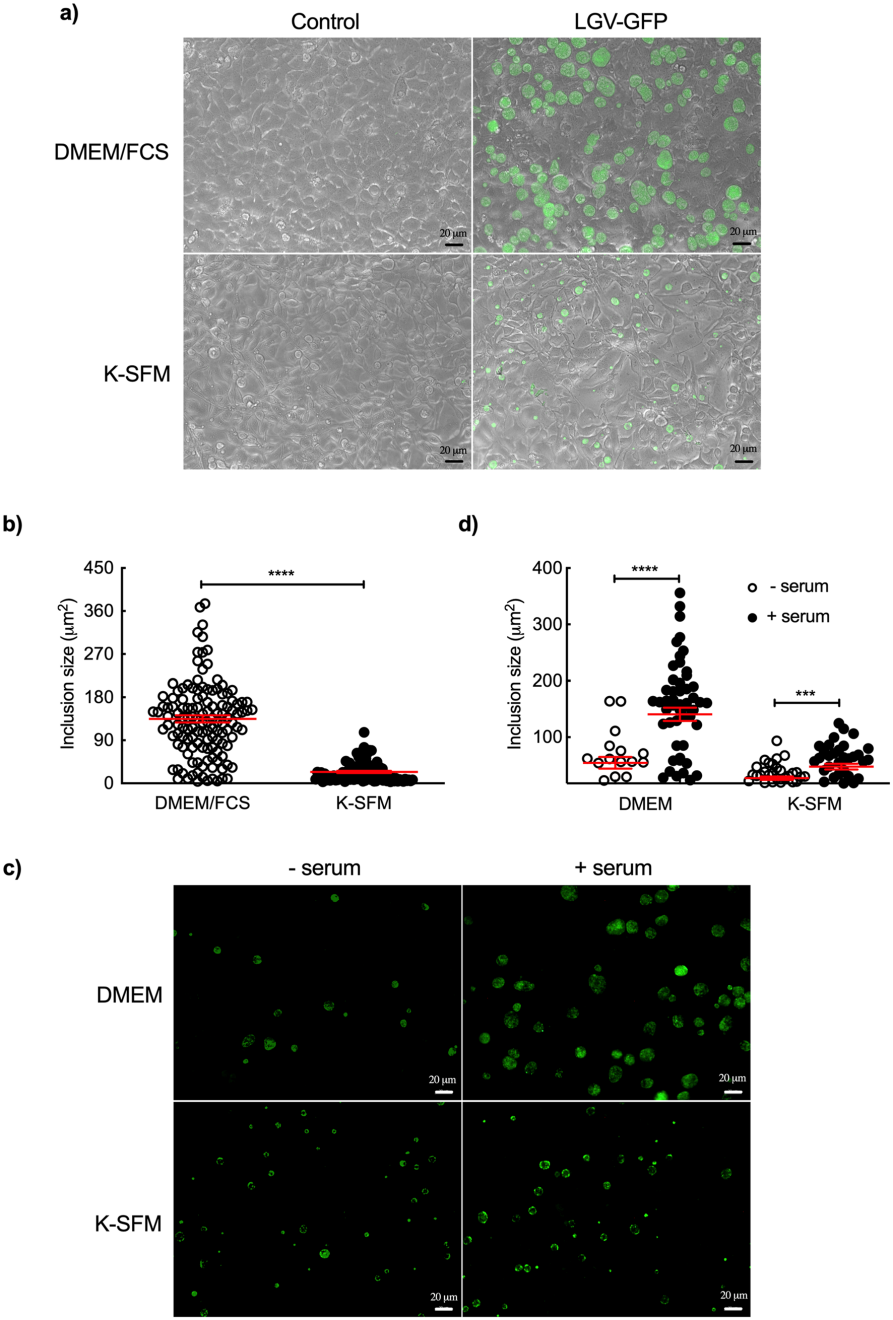
Culture medium and serum affect inclusion size in HeLa cells. (**a**) HeLa cells were incubated in DMEM/FCS or in K-SFM medium and infected (right panels) or not (left panels) with L2^incD^GFP at the same multiplicity of infection. Pictures were taken 40 hpi. (**b**) The dot plot shows the mean area of inclusions in the two conditions, each individual point represents one inclusion. (**c**) HeLa cells were infected with L2^incD^GFP in DMEM (upper panels) or K-SFM (bottom panels) in the presence (left panels) or absence (right panels) of serum for 40 h before taking the images. (**d**) The dot plot shows the mean area of inclusions in the four conditions, each individual point represents one inclusion. Two independent experiments, with inclusions from at least n > 5 images per experiment, were shown to measure the sizes of the inclusions. The data were presented as mean ± SEM and the non-paired Student’s t-test was used for two group comparisons. ****p < 0.0001.

When HeLa cells are infected with *C. trachomatis* at high MOI, multiple inclusions start developing, that then fuse into a single inclusion quickly^22^. Indeed, unique inclusions were observed in most *Chlamydia*-infected HeLa cells (Figs. 2 and 3). Surprisingly, multiple inclusions were regularly observed in *Chlamydia*-infected primary epithelial cells even at low MOI (Fig. 2b,c), and even at late (40 h) time of infection (Fig. 2h). It suggested a delayed inclusion development in primary epithelial cells. One key player in inclusion fusion is IncA, whose absence results in multiple inclusions ^22,23^. IncA was detected in primary epithelial cells, even on multiple inclusions (Fig. S5). This observation indicates that another factor(s) than IncA is limiting in this cellular background to ensure efficient inclusion fusion.

### Differences in culture medium partially account for the differences in *C. trachomatis* development in primary epithelial cells and HeLa cells

To keep their cellular identity primary epithelial cells have to be grown in K-SFM without serum (it is replaced by EGF and BPE), while HeLa cells are grown in DMEM supplemented with FCS. These differences could result in unequal bacterial access to nutrients, and explain the difference of progeny observed between both cell types. To test this possibility, we first compared the size of inclusions, as a read-out for bacterial load, in HeLa cells grown either in DMEM/FCS or in K-SFM. No apparent difference in cell morphology was observed (Fig. 3a), but inclusions were 6 times larger (134 ± 7 vs. 23 ± 2 μm^2^, p < 0.0001) in HeLa cells grown in DMEM/FCS compared to K-SFM (Fig. 3a,b). We next tested the contribution of FCS to these differences. Presence of serum in the culture medium markedly increased *Chlamydia* inclusion size in infected HeLa cells especially when cultured in DMEM (Fig. 3c,d), indicating that FCS contributes significantly to the difference of progeny observed previously. In addition, the differences in inclusion size also depend on the nature of the medium, since inclusions in HeLa cells grown in K-SFM were systematically smaller than in DMEM (27 ± 3 vs. 54 ± 10 μm^2^, p = 0.0017) (Fig. 3c,d).

**Figure 4:**
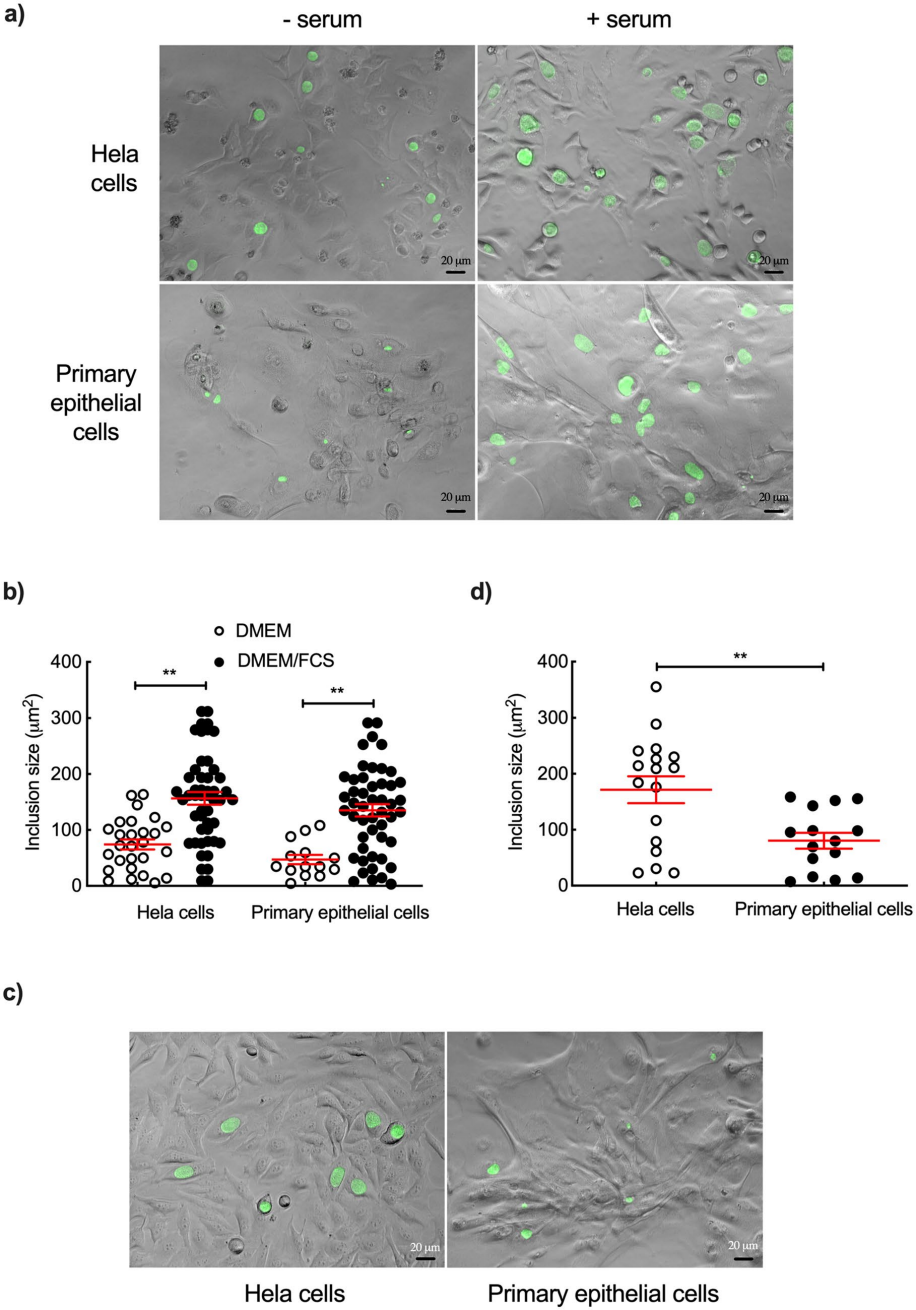
Culture medium and serum affect both cell morphology and inclusion size in primary epithelial cells. (**a**) HeLa and primary epithelial cells were incubated in DMEM medium and infected with L2^incD^GFP at the same multiplicity of infection with (right panels) or without (left panels) serum. Images were taken 40 hpi. (**b**) The dot plot shows the mean area of inclusions in the two conditions for both cell types, each individual point represents one inclusion. (**c**) HeLa and primary epithelial cells were incubated in mixed medium of DMEM/FCS and K-SFM (1:1, v/v) and infected with L2^incD^GFP at the same multiplicity of infection. Pictures were taken 40 hpi. (**d**) The dot plot shows the mean area of inclusions in the two cell types, each individual point represents one inclusion. Three independent experiments, with inclusions from at least n > 5 images per experiment, were performed. The data were presented as mean ± SEM and the non-paired Student’s t-test was used for two group comparisons. **p < 0.01.

We next compared the two cell types cultured in DMEM with or without serum. Addition of serum increased the size of inclusions for the two cell types. In contrast, the difference in inclusion size was modest in the absence of serum between the two cellular backgrounds (Fig. 4a,b). These results indicate that medium composition, and, most importantly, addition or not of serum, significantly affect bacterial growth. However, these data are difficult to interpret because transfer from K-SFM to DMEM induced important changes in the morphology of epithelial cells (Fig. 4a), suggesting that they might no longer behave as *bona fide* epithelial cells.

The last condition chosen to compare the two cell types was to incubate them in mixed DMEM/FCS and K-SFM (ratio 1/1, v/v). As observed in DMEM only (Fig. 4a), primary epithelial cells became more elongated, while the morphology of HeLa cells remained unchanged (Fig. 4c). Inclusions were about two-fold smaller (80 ± 14 vs. 171 ± 24 μm^2^) in primary epithelial cells than HeLa cells (Fig. 4c,d). However, the percentage of infection was similar between HeLa and primary epithelial cells grown in this mixed medium when the same dose of bacterial was used (data not shown). This result shows that, independently of culture medium, *C. trachomatis* development is slower in primary epithelial cells than in HeLa cells.

### Comparison of the immune response from primary epithelial cells and HeLa cells upon *Chlamydia* infection

**Figure 5:**
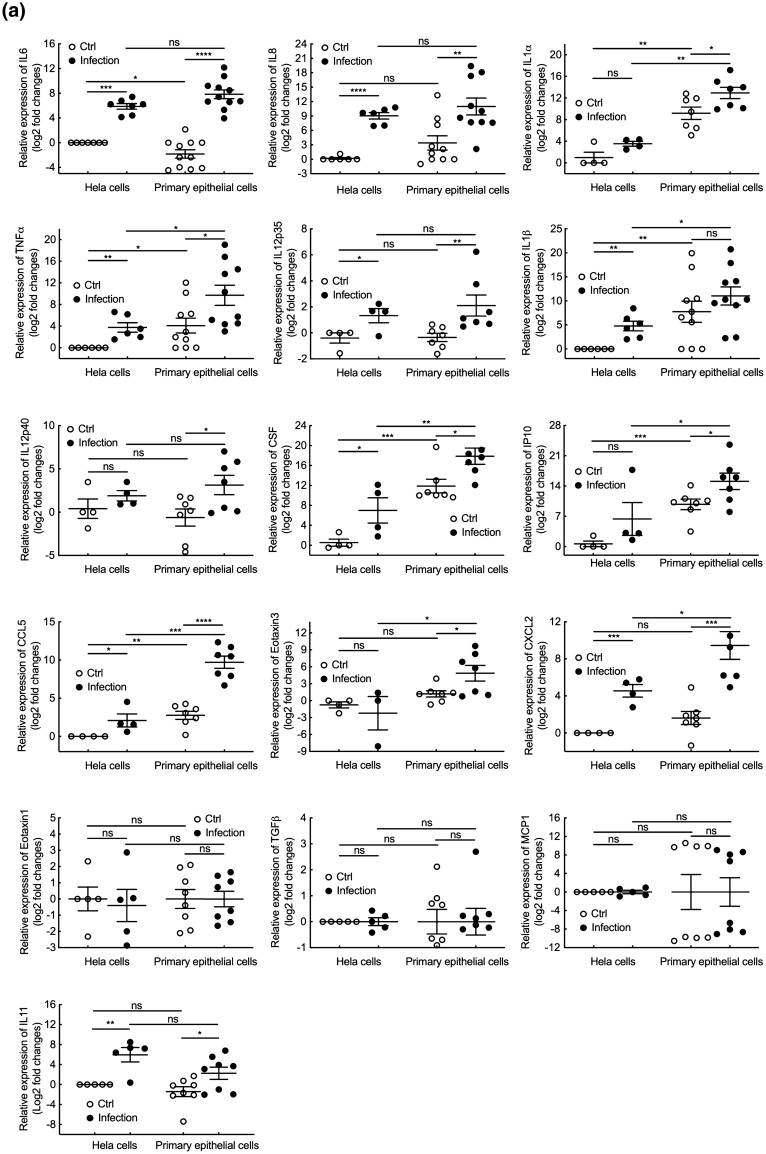

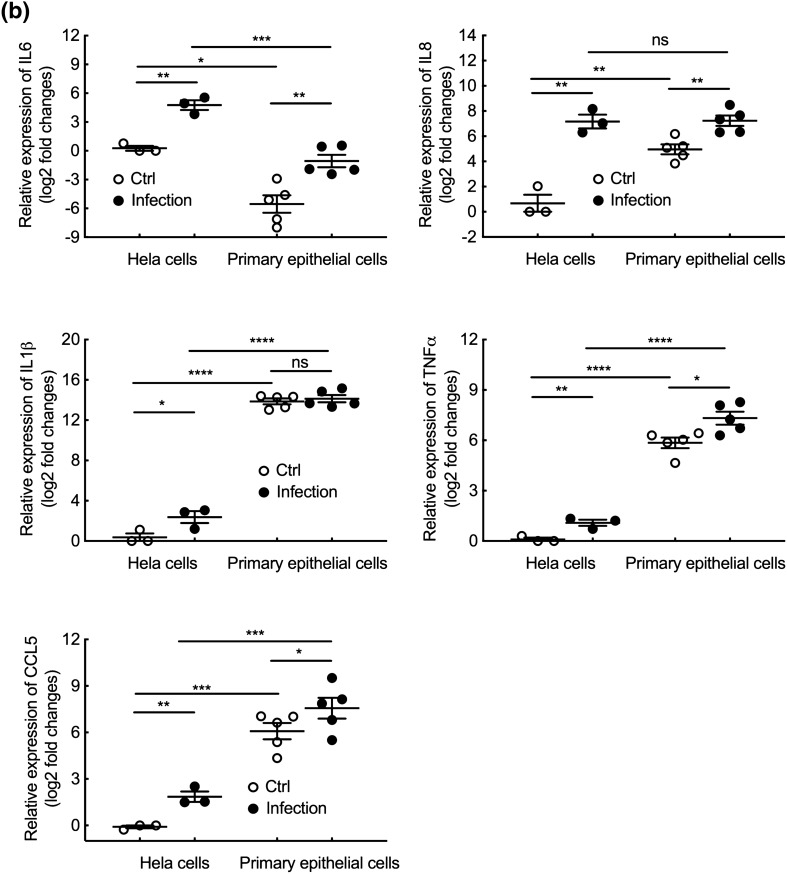
Inflammatory response of HeLa and primary epithelial cells upon *Chlamydia* infection. (**a**) HeLa and primary epithelial cells were infected with *C. trachomatis* serovar L2 (MOI = 1 for HeLa cells and MOI = 10 for primary epithelial cells, respectively). Thirty hours post infection, RNAs were extracted from cell lysates and the transcripts for the indicated genes were measured by real-time quantitative PCR and normalized to house-keeping gene β-actin following the 2^−ΔΔCt^ method. (**b**) HeLa and primary epithelial cells were infected with *Chlamydia* serovar D (bacterial stock diluted 1:500 for HeLa cells and 1:50 for primary epithelial cells). Forty-eight hours post infection, the samples were processed as in (**a**). The data are presented as Log2 fold changes of mRNA compared to uninfected HeLa cells. Each plot displays data obtained from at least 3 independent experiments and each individual point in the primary cell group represents the results of cells isolated from a different donor. The data were presented as mean ± SEM and the non-paired Student’s t-test was used for two group comparisons. *p < 0.05; **p < 0.01; ***p < 0.001; ****p < 0.0001; ns, not significant.

Finally, the inflammatory response of primary epithelial and HeLa cells upon *Chlamydia* infection was examined. Ten times more *C. trachomatis* serovar L2 bacteria were used to infect the primary epithelial cells, to reach 85 to 100% infection in both cellular backgrounds. Transcription levels of four major proinflammatory cytokines, *IL6, IL8, IL1β* and *TNFα*, were examined at different times post infection. We observed a robust inflammatory response in primary epithelial cells, with a peak in transcriptional up-regulation around 24–30 hpi for all cytokines, except for *IL1β,* that showed very modest induction (Fig. S6). Interestingly, transcriptional up-regulation was stronger in primary epithelial cells than in HeLa cells. Based on these results, we next measured transcription levels of various inflammatory cytokines 30 hpi (Fig. 5a). Primary epithelial cells displayed a higher basal level of transcription of genes for a number of proinflammatory cytokines (*IL1α*, *IL1β*, *TNFα*, *CSF*, *IP10* and *CCL5*), compared to HeLa cells, with the notable exception of *IL6*. Infection with *C. trachomatis* serovar L2 induced expression of several pro-inflammatory cytokines, including *IL6*, in both HeLa and primary epithelial cells, and the response was stronger in primary cells (Fig. 5a). For instance, a marked increase of the transcripts for the inflammatory cytokines *IL1α, IL12p40, IP10* and *eotaxin3* was detected in primary epithelial cells upon infection, while, no, or only moderate, increase was observed in the HeLa cell line. We wondered whether the difference could be due to discordant *IL11* response between the two cell types, as this cytokine limits the expression of *TNFα, IL1β, IL6, IL12p40* and *NO* in LPS-stimulated macrophages^24,25^, and was proposed to play a role in limiting the early response to *C. trachomatis* infection^20^. However, basal *IL11* transcript levels were similar between primary epithelial cells and HeLa cells, and showed similar induction by *Chlamydia* infection in the two cellular backgrounds (Fig. 5a). Thus, the difference in inflammatory profiles between the two cell type does not appear to be linked to *IL11* levels. Of note, several proinflammatory cytokines did not respond to *Chlamydia* infection in both cell types, including *eotaxin1, MCP1* and *TGFβ*. Finally, we examined transcripts levels for five inflammatory cytokines, *IL6 IL8, IL1β, TNFα* and *CCL5*, upon infection with *C. trachomatis* serovar D. All cytokines were up-regulated in primary epithelial cells except *IL1β*, consistent with the observations made with serovar L2 (Fig. 5b). However, the amplitude of the response was lower than in cells infected with serovar L2, an observation consistent with the slower proliferation of the serovar D bacteria compared to serovar L2 (Fig. 2b,c). Altogether, these results revealed that primary epithelial cells had a distinct pattern of inflammatory response, and, overall, displayed a stronger inflammatory response to *C. trachomatis* infection than HeLa cells. The high basal level of expression of pro-inflammatory cytokines in primary epithelial cells may contribute to this behavior.

## Discussion

The present study establishes a simple protocol for isolating primary epithelial cells from cervical biopsies of donors. The whole procedure can be performed within 24 h or even less, if the biopsy is collected in the morning. The primary epithelial cells can be passaged 4 to 5 times. The cells expand fast during the first 3 passages, then slow down. After 5 passages, the cells stop dividing in general. In our hand, the first 3 passages of primary epithelial cells could be cryopreserved and grew well once thawed, in contrast to one previous study^17^.

Compared to other methods in which enzymatic cocktails were applied to isolate primary epithelial cells from the fallopian tubes and the uterus of human samples^14,26,27^, a single enzyme Dispase^®^ II was used. A similar methodology was used by other groups in isolating primary cervical epithelial cells^21,28^. One important step we introduced was the mechanical separation of the mucosal layer from the stromal tissue before dissociating the epithelial cells, which significantly improved the purity of isolated cells.

Avoiding contamination by fibroblasts or other cells is a major challenge for the isolation of primary epithelial cells^29^, as contamination of the culture is a common confounding factor. This was achieved by three complementary strategies. Firstly, the mucosal layer containing the epithelia was separated completely from the stromal tissue at an early stage of the procedure. Secondly, a new defined K-SFM medium was introduced, which significantly slowed down fibroblast growth. Lastly, most of the experiments were performed after the second passage of the primary cells, largely reducing the risk of contamination by other cell types. One other commonly used strategy to expand primary epithelial cells from the cervix is to place the small pieces of explant with mucosal surface down directly into the culture dish^11,17,30^. In our hands, the present protocol was less prone to fibroblast contamination.

 Primary epithelial cells isolated from human FGT have been cultured in a range of different media including DMEM/FCS^10^, DMEM/F12^14,15^, keratinocyte growth medium KGM (Clonetics)^18^, RPMI1640/FCS/KGM^17^, K-SFM (Thermo Fisher Scientific)^13^, EpiGRO Human Epidermal Keratinocyte Complete Media (Millipore)^30^ and even lab-made medium^12^. Surprisingly, this is the first report that elongated cell, reminiscent of the morphology of fibroblasts appeared in the culture when primary epithelial cells were switched from K-SFM to DMEM/FCS or to DMEM/FCS mixed with K-SFM. It is unclear whether this reflects a morphological change in the “epithelial” cells, or if contamination became apparent because fibroblasts divide much faster than epithelial cells in this culture medium. Relevant to this observation, serum can promote the growth of fibroblasts and inhibit keratinocytes *in vitro*^31^. Changes in cell morphology and growth upon modification of the culture medium have been also reported, even on cell lines^32^.

Consistent with recent reports^12,17^, *C. trachomatis* was able to infect primary epithelial cells isolated from cervical biopsies. Compared to HeLa cells, we and other^18^ observed a reduction in the percentage of infected cells, and in the size of inclusions, indicating that epithelial cells are less permissive to *Chlamydia* infection than HeLa cells. One confounding factor in this comparison is that different culture media need to be used to maintain these two cell types, which likely affects bacterial supply in nutrients. Our experiments showed that indeed, the presence of serum boosts inclusion growth, whatever the cellular background. However, these experiments are difficult to interpret because we observed that the morphology of epithelial cell was very sensitive to the culture conditions, suggesting that replacement of K-SFM with DMEM is sufficient to change their identity, and possibly their ability to respond to infection. Still, when grown in the same mix of DMEM/FCS and K-SFM, primary “epithelial” cells remained less permissive to *C. trachomatis* development than HeLa cells. This observation indicates that primary epithelial cells have intrinsic anti-chlamydial properties that are defective in HeLa cells, and emphasize the need to use primary cells to study the response of the epithelial layer to infection.

Cell-autonomous immune responses play an important part in the host arsenal to eliminate invading pathogens. Transcripts for several pro-inflammatory cytokines were detected in basal conditions (i.e. non-infected cells), and their level was systematically higher in primary epithelial cells than in HeLa cells. Early work on the constitutive secretion of IL-8 and other proinflammatory cytokines by primary epithelial cells of FGT came to the same conclusion^15,18^. This phenomenon may in part reflect the differences in culture conditions. Infection by *C. trachomatis* elicited an increase of transcription of inflammatory genes in primary epithelial cells. This observation is consistent with the increase in secretion of several inflammatory cytokines and chemokines including IL6, IL8, IL10, Eotaxin3, TNFα, MCP1, MIP1, observed in primary endocervical epithelial cells infected with *C. trachomatis*^17^. The range of the transcriptional response to infection was lower in HeLa cells, and was consistent with transcriptomic data collected in this cell type^33^.

The first cells encountered by *C. trachomatis* upon sexual transmission are the epithelia cells from the lower FGT, the vagina and the ecto-cervix. Robust replication of *C. trachomatis* in immortalized human vaginal epithelial cells was reported recently^16^, and we show here that epithelial cells from the ecto-cervix are also permissive to *C. trachomatis* growth. Other work on primary cells have focused on columnar cells isolated from the endocervix^34^. Interestingly, no significant increase in proinflammatory cytokines including IL6, IL8 and TNFα was observed in this cellular background^34^, in contrast to what we observed in ectocervical primary cells, indicating that the ectocervix might be better equipped to alert the surrounding environment of an intrusion than the endocervix.

In conclusion, the present study demonstrated a slower rate of *Chlamydia* replication in primary epithelial cells isolated from human ectocervical biopsies than in HeLa cells, although this is largely due to difference in culture medium. The most important difference we report here is the stronger ability of ectocervical epithelial cells to deploy an inflammatory response compared to the commonly used HeLa cell line. This support the hypothesis that epithelial cells, while representing the target of *C. trachomatis* replication, also constitute a well-equipped line of defense to counteract the spread of the pathogen. More studies using primary epithelial cells will be required in the future to understand which level of protection this response offer, and why it is sometimes insufficient to contain the ascension of *C. trachomatis* to the upper FGT.

## Methods

### Collection of samples and ethical approval

All methods to recruit subjects and collect biological samples were carried out in accordance with relevant guidelines and regulations. All participants (ages ranging from 38 to 55, in pre-menopause), gave written informed consent in accordance with the Declaration of Helsinki principles. Data and samples were collected from women undergoing hysterectomies due to different gynecological problems including uterine fibroma, endometriosis, prolapse of the uterus and adenomyosis. The clinical study was approved by the French Ethical Committee ‘CPP Ile de France 1′ on May 9, 2016. Approval and authorization of the National Data Protection authority (‘Commission Nationale de l’Informatique et des Libertés’, CNIL) have been obtained for the research protocol. Ecto-cervical explants distal from the pathological site were rinsed in RPMI 1640 (Thermo Fisher Scientific) containing gentamycin (5 μg/ml), and stored in this medium at 4 °C until transport to the laboratory (within 2 to 18 h after surgery).

### Isolation of primary epithelial cells and fibroblasts

The explant was rinsed several times with pre-chilled PBS containing gentamycin (5 μg/ml) to remove the blood. The non-mucosal tissues, such as muscle and stromal tissue, were removed as much as possible under a binocular microscope. The remaining part containing the mucosal layer (1–1.5 cm^2^) was cut into small pieces (around 3 × 3 mm^2^) and placed, mucosal surface face-down, in a 6-well culture dish containing Dispase^®^ II (Roche) solution (2.4 units/mL in RPMI 1640).

Dispase^®^ II digestion was performed either at 4 °C overnight or at 37 °C for 3–4 h. After the digestion, the mucosal layers were separated from the stromal tissue using forceps, and pooled together into a 15 ml centrifugation tube containing RPMI 1640. The mucosal layers were pelleted by centrifugation (500×*g* for 5 min at room temperature) and re-suspended with 0.25% Trypsin, 0.02% EDTA solution in Hanks′ Balanced Salt Solution (Sigma-Aldrich) and digested further for 10 min at 37 °C. Digestion by trypsin was stopped by adding fetal calf serum (FCS). After vigorous vortex, the suspension was passed through a 40 μm mesh to get rid of the non-digested debris. The collected cell suspension was pelleted by centrifugation (2000×*g* for 15 min at 4 °C). The pellet was resuspended into Gibco™ keratinocyte serum-free media supplemented with 5 µg/L of human recombinant epidermal growth factor (EGF) and 50 mg/L bovine pituitary extract (BPE) (K-SFM, Thermo Fisher Scientific, #17005075), and seeded in 75 cm^2^ culture flasks. The culture medium was changed every 2–3 days until cell reached confluency (10–15 days depending on the mucosal size of the explant). Upon confluency cells were rinsed twice by 0.5 mM EDTA in PBS, detached by incubation with 1 mL of Trypsin/EDTA at 37 °C for 5 min. The cell suspension and rinsing medium were pooled together and centrifuged again. The pellet was resuspended in K-SFM and re-plated in 150 cm^2^ dishes for further expansion (dilution 1:2 to 1:4). The cells at passage 2–3 were used for the experiments or cryo-frozen in K-SFM and DMSO suspension (1:1, v:v) containing around 1 × 10^6^ cells per vial. The data presented in Fig. S1 were obtained using a novel culture medium, the Gibco™ defined K-SFM (Thermo Fisher Scientific, #10744019). The rest of the data were obtained with early passage (P2) epithelial cells cultured in K-SFM.

For isolation of fibroblasts, the cells isolated as described above were cultured in Dulbecco’s modified Eagle’s medium with Glutamax (DMEM, Invitrogen) containing 10% fetal calf serum (FCS). After 2–3 passages, the large majority of cells were fibroblasts and either used for the experiment or cryo-frozen. HeLa cells (ATCC) were cultured in DMEM medium containing 10% FCS.

### Bacterial preparation and infection

*Chlamydia trachomatis* serovar L2 (434/Bu) and sevevar D (UW-3/CX) strains were propagated in HeLa cells. In certain experiments, a L2 strain stably expressing the green fluorescent protein under the control of the IncD promoter (L2^incD^GFP) was used^35,36^. The purified elementary bodies (EBs) of *Chlamydia* were stored in sucrose-phosphate-glutamic acid buffer (SPG) at − 80 °C.

For infection, adhered cells (0.2 × 10^6^ cells/well in 12-well plates) were washed twice in serum-free medium and incubated with bacteria for the indicated time before collecting the samples. For serovar D infection, the plates were centrifuged at 1200×*g* for 30 min at 30 °C to facilitate the infection.

### Adhesion assay

Adhesion assays were performed as previous description^36^. Briefly, HeLa or primary epithelial cells seeded in a 24-well plate the day before (0.1 × 10^6^ cells/well) were pre-cooled 30 min at 4 °C and then incubated for 4 h at 4 °C with L2^IncD^GFP (MOI = 10). The bacteria were sonicated prior infection to disrupt bacterial aggregates. After incubation, cells were washed gently with PBS 3 times and detached using 0.5 mM EDTA in PBS. The pelleted samples were fixed in 4% (w/v) paraformaldehyde (PFA) 4% (w/v) sucrose in PBS for 30 min, washed with PBS and analyzed using flow cytometry.

### Bacterial entry assessment

Entry experiments were performed as described previously^36^. In brief, HeLa or primary epithelial cells seeded on coverslips in a 24-well culture dish the day before (0.1 × 10^6^ cells/well) were pre-cooled 30 min at 4 °C and then incubated for 45 min at 4 °C with L2^IncD^GFP bacteria (MOI = 10). The bacteria were sonicated before infection to disrupt bacterial aggregates. After incubation, cells were rinsed with pre-chilled PBS. Then pre-warmed medium was added and incubated at 37 °C before being fixed at different time points in 4% PFA 4% sucrose in PBS for 20 min. Extracellular bacteria were stained with a mouse anti-MOMP-LPS antibody (Argene #11–114) followed with Cy5-conjugated anti-mouse secondary antibody (Amersham Biosciences). The dilutions were made in PBS containing 3% of BSA. DNA was stained using 0.5 µg/mL of Hoechst 33342 (Thermo Fisher Scientific) added in the secondary antibody solution. Images were acquired on a DeltaVision Elite microscopy system (GE Healthcare Life Science), equipped with an Olympus IX71 inverted microscope (Olympus, Japan). Images were taken with a CoolSnap HQ2 cooled CCD camera using the software Zen. The bacterial entry was presented as ratio between intracellular bacteria (single GFP staining) and total bacteria (all GFP positive staining including GFP/Cy5 double stained bacteria).

### Flow cytometry

After infection or treatment, the cells were detached with 0.5 mM EDTA in PBS and transferred to a Falcon™ 96-well U-bottom microplate (Corning, USA) for centrifugation (500×*g* for 5 min). The pellets were resuspended in 4% PFA 4% sucrose in PBS for 20 min fixation at room temperature. The cells were washed with PBS and incubated with 50 mM NH_4_Cl to quench the residual PFA. After blocking in 1% BSA in PBS for 15 min, the samples were incubated in PBS, 0.1% BSA with or without 1/200 dilution of FITC conjugated anti-human E-cadherin (BD Sciences, #612130) for 1 h. After washes, the cells were resuspended in PBS and analyzed by CytoFLEX flow cytometer (Beckman Coulter). For experiments using L2^IncD^GFP, the cells were fixed and analyzed by flow cytometry directly. The data were analyzed using FlowJo (version 10.0.7).

### Cell imaging and measurement of inclusion size

The cells (0.1 × 10^6^ cells/well) seeded on coverslips in a 24-well culture dish were infected with L2^IncD^GFP strain or with *C. trachomatis* serovar D strain. Tenty-four to 40 h later, the cells were fixed with 4% PFA 4% sucrose for 20 min at room temperature. After fixation, the cells were washed in PBS and incubated for 10 min in 0.05% (w/v) saponin, 0.1% (w/v) BSA and 0.5 µg/mL of Hoechst 33342 (Thermo Fisher Scientific) to enable cell permeabilization and DNA staining. Coverslips were mounted on slides in a Mowiol solution. Serovar D-infected cells were stained with antibody against *Chlamydia* Cap1 and A488-conjugated secondary antibody. Images were acquired on an Axio observer Z1 microscope equipped with an ApoTomemodule (Zeiss, Germany) and a 63 × Apochromat lens. Images were taken with an ORCAflash4.OLT camera (Hamamatsu, Japan) using the software Zen. For characterizing epithelial cells, the fixed primary cells were incubated for 1 h in PBS, 0.1% (w/v) BSA and anti-human E-cadherin (1/200), followed with Cy5 conjugated anti-rat (1/500) secondary antibody, or anti-human cytokeratin 14 (GenTex, #GTX104124) and 18 (Invitrogen, #MA5-12104) followed with corresponding Cy5- and A488-conjugated secondary antibodies for an additional hour. DNA was labeled with 0.5 µg/mL of Hoechst 33342 (Thermo Fisher Scientific). IncA was stained with rabbit anti-IncA antibodies (kind gift from T. Hackstadt, Rocky Mountain Laboratories). In certain experiment, the cells were fixed directly in the culture dish and observed under an Axio Observer microscope (Zeiss, Germany). The size of inclusions (μm^2^ in area) was measured using ImageJ software. All inclusions from at least 5 fields were counted.

### Progeny assay

One hundred thousand cells were seeded in a 24-well plate. The next day cells were infected with L2^IncD^GFP bacteria at a MOI = 0.15 for HeLa cells, and with ten to twenty times more bacteria for primary epithelial cells, in order to reach about 15% infected cells in both cell types (this factor was later considered for the calculation of the progeny). Forty-eight hpi, cells were detached, lysed using glass beads and the supernatant was used to infect HeLa cells plated the day before (0.1 × 10^6^ cells/well in a 24-well plate), in serial dilutions. Twenty-four hours later, cells with an infection lower than 30% (checked by microscopy) were detached and fixed as described above, before analysis by flow cytometry and determination of the bacterial titer.

### RT-PCR and quantitative PCR

The adhered cells (0.1 × 10^6^ cells/well) in 24-well plate were infected or not with *C. trachomatis* serovar L2 or D (MOI = 1 for HeLa cells and MOI = 10 (titrated in HeLa cells) for primary epithelial cells). Total RNAs were isolated 6 to 40 hpi for serovar L2 infection and 48 hpi for serovar D infection with the RNeasy Mini Kit (Qiagen) and RNA concentrations were determined with a spectrophotometer NanoDrop (Thermo Fisher Scientific). Reverse transcription (RT) was performed using the M-MLV Reverse Transcriptase (Promega) and real-time quantitative PCR (qPCR) undertaken in triplicates on the complementary DNA (cDNA) with LightCycler 480 system (Roche) using SYBR Green Master I (Roche). Data were analyzed using the 2^−ΔΔCt^ method with *actin* as a control gene and results were presented as fold changes compared to non-infected control. The primers were purchased from Eurogentec and are shown in Table S1.

### Statistical analysis

The experimental data were recorded and analyzed using Prism8 (GraphPad). The non-paired Student’s t-test was used for two group comparisons. P-values less than 0.05 were considered as significant.

## Supplementary Information

Supplementary Figure Legends.

Supplementary Figure S1.

Supplementary Figure S2.

Supplementary Figure S3.

Supplementary Figure S4.

Supplementary Figure S5.

Supplementary Figure S6.
