# Supplementary material for: Primary ectocervical epithelial cells display lower permissivity to Chlamydia trachomatis than HeLa cells and a globally higher pro-inflammatory profile
Source: Sci Rep. 2021 Mar 12;11:5848. doi: 10.1038/s41598-021-85123-7 (PMC7955086; doi:10.1038/s41598-021-85123-7)
Supplement: Supplementary file 1 — Supplementary Figure Legends. [file 41598_2021_85123_MOESM1_ESM.docx]

**Primary ectocervical epithelial cells display lower permissivity to *Chlamydia trachomatis* than HeLa cells and a globally higher pro-inflammatory profile**

Chongfa Tang^1,2,3#^, Chang Liu^1#^, Benoit Maffei^1,3^, Béatrice Niragire^1^, Henri Cohen^4^, Aminata Kane^5^, Anne-Claire Donnadieu^4^, Yael Levy-Zauberman^4^, Thomas Vernay^1^, Juliette Hugueny^1^, Etienne Vincens^5^, Christine Louis-Sylvestre^4^, Agathe Subtil^1*^, Yongzheng Wu^1*^

1 Unité de Biologie cellulaire de l'infection microbienne, Institut Pasteur, UMR3691 CNRS, F-75015, Paris, France.

2 National Vaccine and Serum Institute, Beijing, China

3 Sorbonne Université, Collège doctoral, F-75005 Paris, France

4 Service de Chirurgie gynécologique, Institut Mutualiste Montsouris, F-75014 Paris, France

5 Service de Gynécologie, Clinique Saint Jean de Dieu, F-75007 Paris, France.

Table S1 : Primers used for real-time quantitative PCR

|  |  | sense | anti-sense |
| --- | --- | --- | --- |
| 1 | actin | GGACTTCGAGCAAGAGATGG | GCAGTGATCTCCTTCTGCATC |
| 2 | IL1α | AGATGCCTGAGATACCCAAAACC | CCAAGCACACCCAGTAGTCT |
| 3 | IL1β | CCTCTCTCTAATCAGCCCTCTG | GTCGGAGATTCGTAGCTGGA |
| 4 | IL6 | CACACAGACAGCCACTCACC | CATCCATCTTTTTCAGCCATC |
| 5 | IL8 | AGCCTTCCTGATTTCTGC | GCCCTCTTCAAAAACTTCTC |
| 6 | GM-CSF | TCCTGAACCTGAGTAGAGACAC | TGCTGCTTGTAGTGGCTGG |
| 7 | TNFα | GAGGCCAAGCCCTGGTATG | CGGGCCGATTGATCTCAGC |
| 8 | TGFβ1 | CTAATGGTGGAAACCCACAACG | TATCGCCAGGAATTGTTGCTG |
| 9 | MCP1 | CAGCCAGATGCAATCAATGCC | TGGAATCCTGAACCCACTTCT |
| 10 | CCL5 | CCAGCAGTCGTCTTTGTCAC | CTCTGGGTTGGCACACACTT |
| 11 | CXCL2 | GGCAGAAAGCTTGTCTCAACCC | CTCCTTCAGGAACAGCCACCAA |
| 12 | IL12p35 | CCTTGCACTTCTGAAGAGATTGA | ACAGGGCCATCATAAAAGAGGT |
| 13 | IL12p40 | TGACACCCCTGAAGAAGATG | TTTTGCCAGAGCCTAAGACC |
| 14 | IP10 | GTGGCATTCAAGGAGTACCTC | TGATGGCCTTCGATTCTGGATT |
| 15 | Eotaxin1 | CTGAGGGGGACAAAGATGG | GGGAATAGAGGGCTGGAATG |
| 16 | Eotaxin3 | ATCAGGCAGGAGGAGTTTG | AGCAGCAGGTCTTGGATATG |
| 17 | IL11 | GACCTACTGTCCTACCTGCG | AGTCTTCAGCAGCAGCAGTC |

**Legends to the supplementary figures**

Fig. S1. Key steps in the isolation of primary epithelial cells. a) A cervical explant was cut into small pieces and digested with Dispase II. b) The mucosal layer was mechanically teared off after enzymatic digestion. c-d) Mucosal layers were pooled together and pelleted by centrifugation. e) Trypsin/EDTA-treated cell suspension was passed through a 40 μm cell strainer to remove debris. f) Cell pellet containing epithelial cells.

Fig S2. Defined K-SFM medium inhibits culture contamination by fibroblasts. a) One thawed vial of primary epithelial cells was split into two parts and grown either in K-SFM or in defined K-SFM. b-c) Eleven days after culture, these cells were then re-plated in the indicated culture medium. The morphology of the cells for each culture condition was monitored at regular intervals over 8 days. Two independent experiments were performed.

Fig. S3. Representative example of raw data for bacterial entry experiments. Cells were incubated with L2^incD^GFP bacteria for 30 min at 4°C, before transfer to 37°C. At the indicated times the cells were fixed and external bacteria were labeled with antibody against *Chlamydia* MOMP and Cy5-conjugated secondary antibody. Internalized bacteria (GFP positive) and extracellular bacteria (GFP and Cy5 double positive) appear in green and yellow, respectively.

Fig. S4. Quantification of the inclusion sizes on primary epithelial cells infected with *C. trachomatis* serovar D and stained as in Fig. 2c. Two independent experiments were performed using primary epithelial cells from different donors. Each individual point represents one inclusion (n>5 fields per experiment). ****p<0.0001.

Fig. S5. Expression of IncA in primary epithelial cells. Primary epithelial cells were infected with *C. trachomatis* serovar L2. At the indicated times post infection, cells were fixed and stained with antibody against the bacterial inclusion protein IncA and A488-conjugated secondary antibody. DNA was labeled with Hoechst. The images are representative of observations made using cells from 4 different donors.

Fig. S6. Kinetics of *Chlamydia*-induced inflammatory response in HeLa and primary epithelial cells. HeLa and primary epithelial cells were infected with *C. trachomatis* serovar L2 (MOI=1 for HeLa cells and MOI=10 for primary epithelial cells, respectively). At the indicated times post infection, RNAs were extracted from cell lysates and the transcripts for the indicated genes were measured by real-time quantitative PCR and normalized to *β-actin* following the 2^-ΔΔCt^ method. The data were presented as Whiskers boxplot in Log2 fold changes of mRNA compared to uninfected HeLa cells. Each plot displays data obtained in at least 3 independent experiments and each individual time-point in the primary cell group represents the results in cells isolated from at least 4 donors. *p<0.05, **p<0.01, ***p<0.001 and ****p<0.0001, compared to corresponding control. ns, not significant.
