# Supplementary figures and images for: Primary ectocervical epithelial cells display lower permissivity to Chlamydia trachomatis than HeLa cells and a globally higher pro-inflammatory profile
Source: Sci Rep. 2021 Mar 12;11:5848. doi: 10.1038/s41598-021-85123-7 (PMC7955086; doi:10.1038/s41598-021-85123-7)

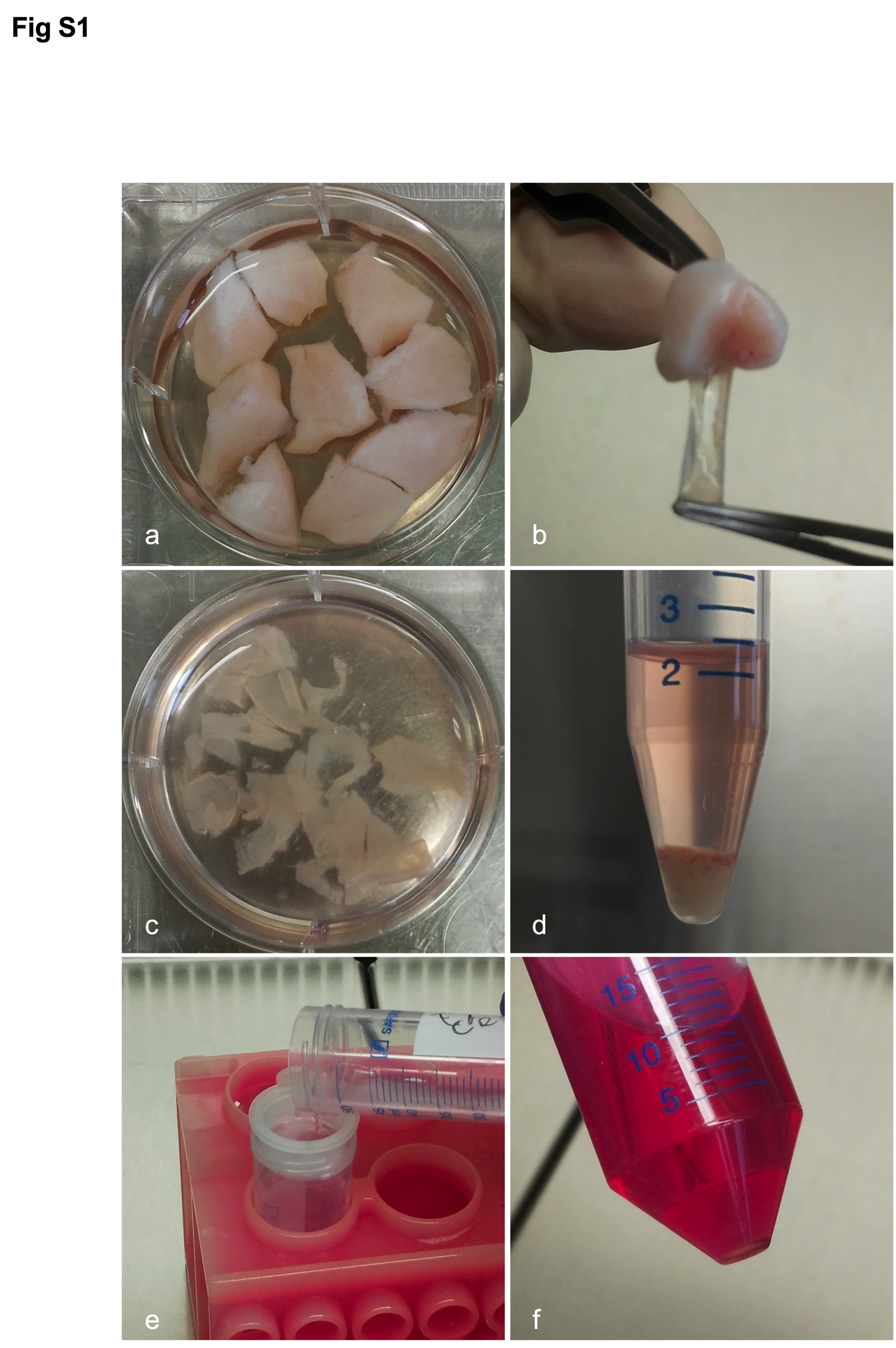

Supplement: Supplementary file 2 — Supplementary Figure S1. [file 41598_2021_85123_MOESM2_ESM.tiff]

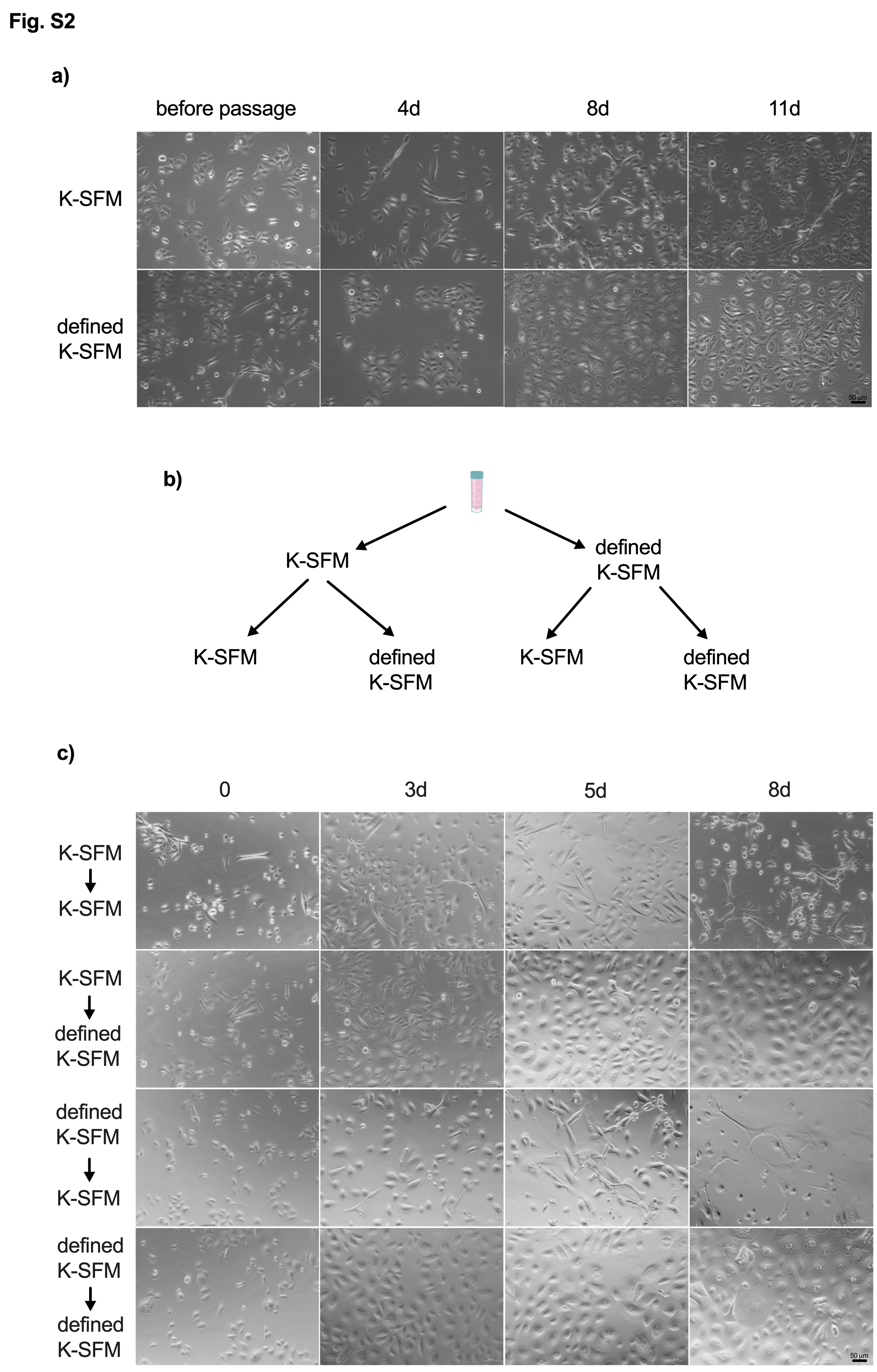

Supplement: Supplementary file 3 — Supplementary Figure S2. [file 41598_2021_85123_MOESM3_ESM.tiff]

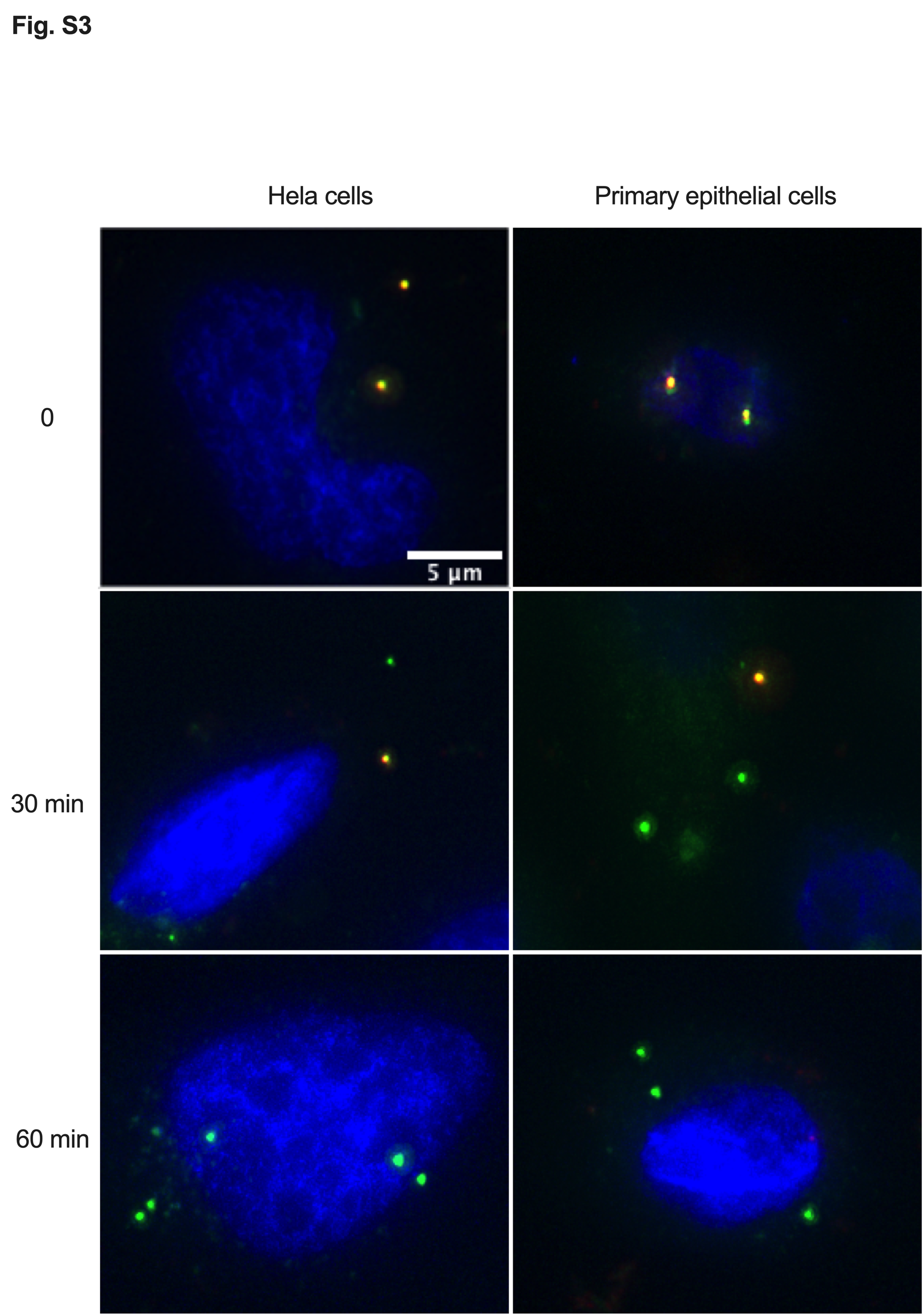

Supplement: Supplementary file 4 — Supplementary Figure S3. [file 41598_2021_85123_MOESM4_ESM.tiff]

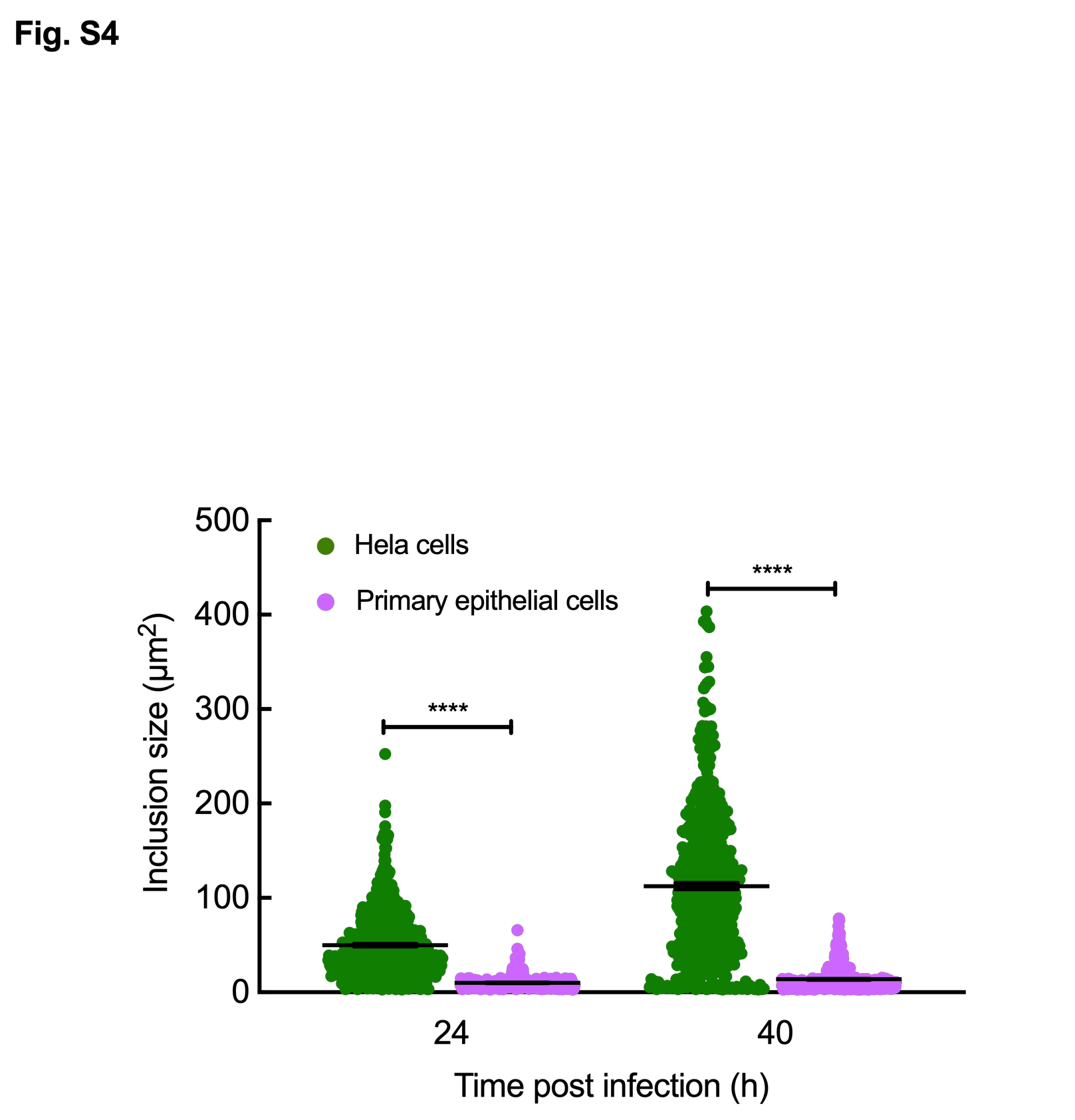

Supplement: Supplementary file 5 — Supplementary Figure S4. [file 41598_2021_85123_MOESM5_ESM.tiff]

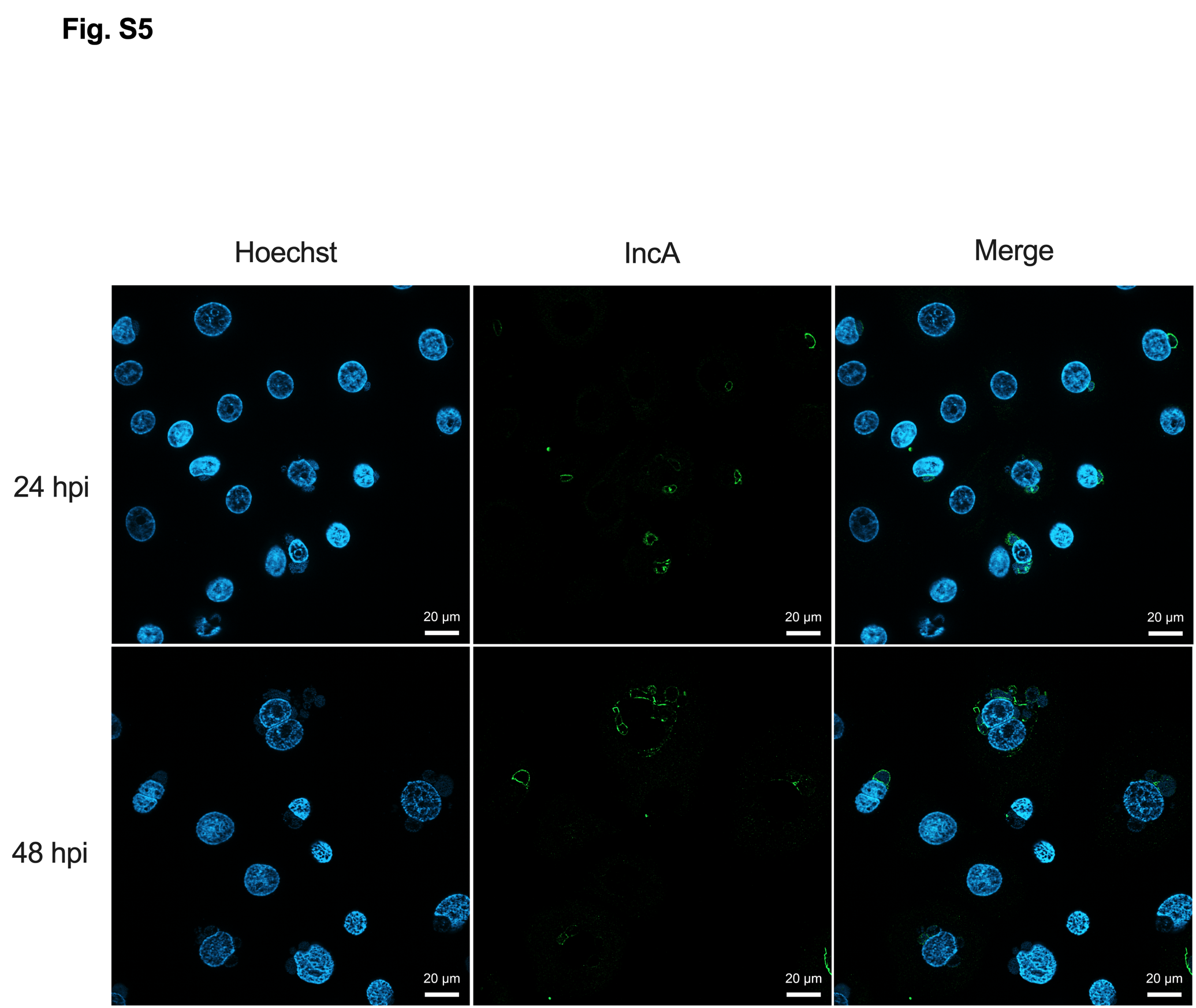

Supplement: Supplementary file 6 — Supplementary Figure S5. [file 41598_2021_85123_MOESM6_ESM.tiff]

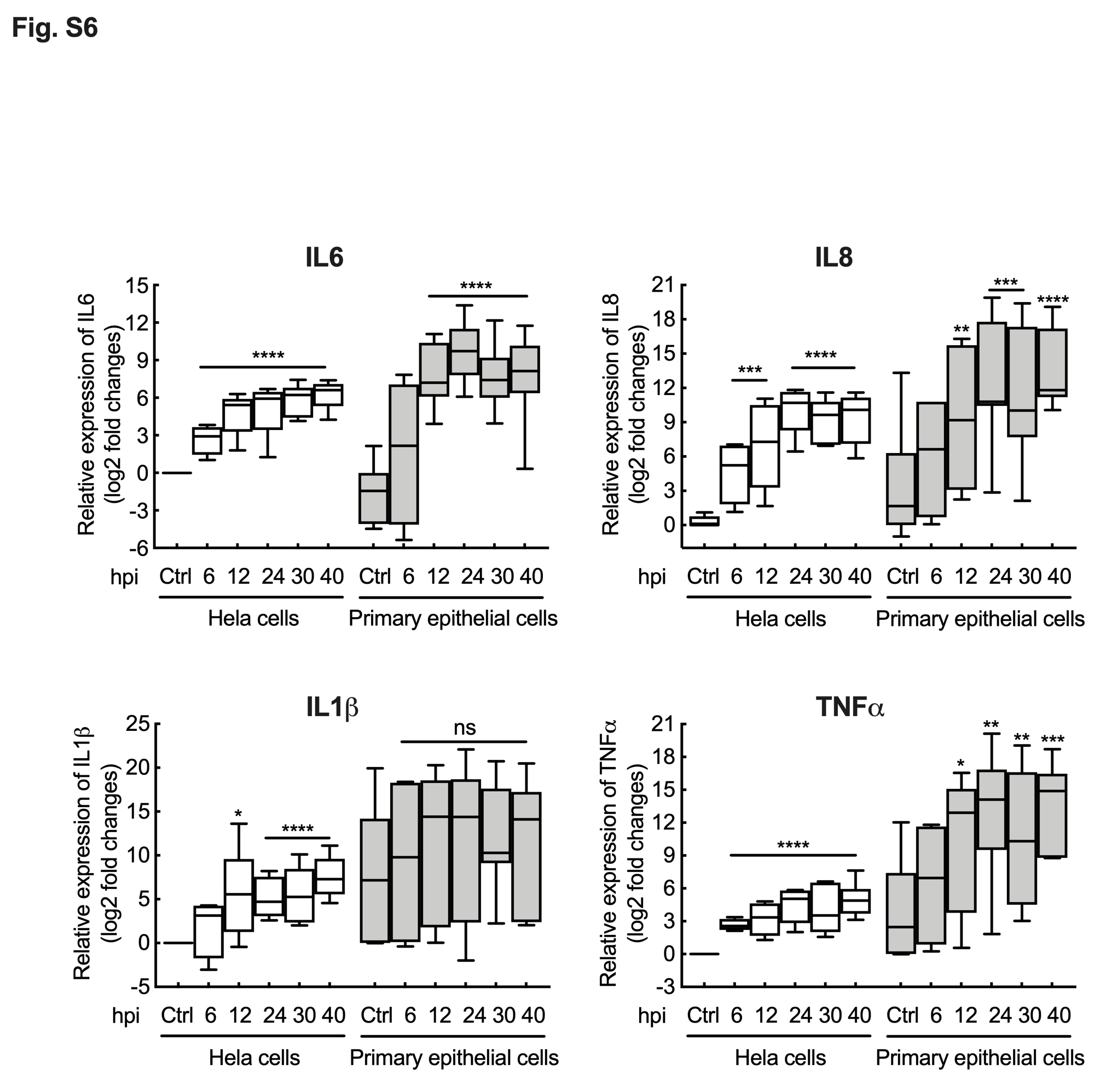

Supplement: Supplementary file 7 — Supplementary Figure S6. [file 41598_2021_85123_MOESM7_ESM.tiff]
